# Comparison of the Allelopathic Potential of Non-Native and Native Species of Mediterranean Ecosystems

**DOI:** 10.3390/plants12040972

**Published:** 2023-02-20

**Authors:** Natividad Chaves Lobón, Marisa González Félix, Juan Carlos Alías Gallego

**Affiliations:** Department of Plant Biology, Ecology and Earth Sciences, Faculty of Science, University of Extremadura, 06006 Badajoz, Spain

**Keywords:** allelopathy, invasive species, native species

## Abstract

Allelopathy is a frequent interaction between species in Mediterranean ecosystems and it is also one of the proposed strategies to explain the colonisation of invasive species. To confirm the importance of allelopathic potential as a mechanism of invasion of non-native species in Mediterranean ecosystems, it would be advisable to compare the allelopathic effects of non-native plants with native plants on the same target species and thus avoid overestimating the role of phytotoxicity in the invasion process. The main objective of this work was to compare the allelopathic activity of native species typical of Mediterranean ecosystems, classified as allelopathic, with the allelopathic activity of non-native species that may have an invasive character in these ecosystems. To this end, we selected three native species (*Cistus ladanifer*, *Pistacia lentiscus*, and *Pistacia terebithus*) and three non-native species (*Acacia dealbata*, *Acer negundo,* and *Salix babylonica*), and we analysed their effect on the species *Lactuca sativa* and the native species *Lavandula stoechas* and *Echium plantagineum*. The tests on *L. sativa* showed that all species have allelopathic activity. The tests on *L. stoechas* and *E. plantagineum* revealed that *P. terebinthus* exerted the greatest effect, being the only species that maintained an inhibitory effect at extract concentrations of 50% and 25% in all the analysed parameters, except in germination and cotyledon emergence for *E. plantagineum*. There were no significant differences in the effect on germination between non-native and native species, although significant differences were found in the effect on root size in the three analysed concentrations, with the native species producing greater inhibition. In conclusion, these species exert a negative effect on the selected native target species, but the negative effect of the native species is greater than that of the non-native species. These results indicate that it is important to compare the allelopathic effects of invasive and native species to correctly estimate the phytotoxic effect of invasive species on their invasiveness

## 1. Introduction

Mediterranean ecosystems are characterised by an important biodiversity pool derived from specific climatic conditions, different origins of the flora, and the heterogeneity in the habitat [1]. Species in these ecosystems are subjected to stressful conditions such as fire, high temperatures, herbivory, and water stress [2], and they have developed diverse strategies for protection against these stressors such as the production of secondary metabolites. These compounds have a variety of roles in the life of plants: they act as a defence against predators and pathogens [3,4,5], provide protection against ultraviolet radiation [6,7], enhance the attraction of pollinators [8,9], and acts as allelopathic agents [10,11,12]. Allelopathy is defined as a process of plant–plant chemical interaction with either positive or negative effects [13]; it is not an isolated event in nature and allelopathy can be regarded as a natural strategy protecting plants against environmental ‘‘enemies’’ and competing plants [14]. It is known that stress factors stimulate the production of allelochemicals [15,16], and thus the typical species of Mediterranean ecosystems show an abundant production of these compounds [17,18,19], which are responsible for the allelopathic activity attributed to many of the species of these ecosystems [20,21,22,23,24,25].

On the other hand, allelopathy has been proposed as an interaction involved in the colonisation processes of invasive species [26], in which the allelochemicals released by the invasive species have a negative effect on the germination, growth, and/or reproduction of native species of the invaded community [27]. The relationship between allelopathy and the invasive capacity of species has been the focus of different studies [28], among which it is worth highlighting the meta-analysis conducted by Kalisz et al. [29], who reviewed the allelopathic capacity of 524 different invasive species. The results of the mentioned meta-analysis revealed that 51.4% of the invasive species considered were allelopathic, and that this capacity was not phylogenetically preserved, but it was widely distributed among the different families of invasive species. This phenomenon of invasion attributed to allelopathy is particularly defended when the invaders produce evolutionarily novel chemicals [30,31], which is the basis of the novel weapons hypothesis (NWH). This hypothesis, formulated by Callaway and Ridenour [32], proposes the relationship between invasiveness and allelopathy since invasive plants can release chemical compounds that are new to the autochthonous species, as they did not coexist before; therefore, native species did not co-evolve or develop adequate defense mechanisms to the chemical compounds of non-native species [29,30,31]. Different studies have shown native species suffered more negative effects from invasive species (due to the concentration or proportion of allelochemicals) than from native species [33,34,35,36,37,38].

However, there are other studies that did not find differences in the allelopathic behaviour between invasive and native species [39,40,41,42], suggesting that it depends on the invasive species and on the characteristics of the native species that are present in the invaded habitat. The characteristics of the different ecosystems determine the more or less allelopathic nature of the species that constitute it, and this could influence the displacement of native species by invasive species.

In ecosystems such as the Mediterranean, with an abundance of species classified as allelopathic [20,21,22,23,24,25], the native plants may also interact with other plants through allelopathy. To confirm the importance of allelopathic potential as a mechanism of invasion of non-native species in these ecosystems, it would be advisable to compare the allelopathic effects of non-native plants with native plants on the same target species and thus avoid overestimating the role of phytotoxicity in the invasion process [41]. Therefore, the main objective of this work was to compare the allelopathic activity of native species that are typical of and widely distributed in Mediterranean ecosystems with the allelopathic activity of non-native species that may have an invasive character in these ecosystems, quantifying their phytotoxicity on the target species. To this end, three native species (*Cistus ladanifer*, *Pistacea lentiscus*, *and Pistacea terebinthus)* and three non-native species (*Acacia dealbata*, *Acer negundo*, and *Salix babylonica*) were selected.

*Cistus ladanifer* is a shrub species of the family Cistaceae. It is distributed in the Western Mediterranean region, from Portugal and Morocco to the French Riviera and Algeria [43]. This species is rich in phenols and terpenes, and it is classified as an allelopathic species [25,44]. The other two native species selected for this study belong to the genus *Pistacea* (*P. lentiscus* and *P. terebinthus*) and they are shrubs of the family Anacardiaceae that live in the Mediterranean region [45]. These species are rich in phenols [46,47,48] and have been attributed to a high allelopathic potential [49,50,51]. The non-native species were selected based on the list of invasive species published by Blanco et al. [52]. *Acacia dealbata* is an evergreen tree of the family Fabaceae. It is from Southeastern Australia and Tasmania [53] and has been widely distributed in the Iberian Peninsula [54]. Different studies have demonstrated that this species exerts a negative effect on the germination of other plants, with its allelopathic activity being considered a mechanism of its invasive capacity [55,56,57,58]. Another non-native species selected was *Acer negundo*, which is a tree of the family Sapindaceae. This species comes from Central and North America [59]. In the Iberian Peninsula, as in other parts of the world, it has become an invasive [60] or potentially invasive species [51]. Other studies have shown its negative effect on the germination and growth of other species, thus regarding allelopathy as a possible mechanism of its invasive capacity [60,61]. The third species selected was *Salix babylonica*. This is a deciduous tree of the family Salicaceae, which comes from Central and Southern Asia [62]. In Europe, it has become an ornamental plant and, more recently, it is also being used for riverbank fixation, becoming a potentially invasive species in many regions [63]. It has been demonstrated that the phenolic compounds of its leaves could have a negative effect on the soil microbiome [64].

The two native species used as target species were *Lavandula stoechas* (woody species) and *Echium plantagineum* (herbaceous species) which share their habitat with the selected species [65,66].

## 2. Results

### 2.1. Total Phenol and Flavonoid Amount

The quantification of total phenols and flavonoids in each of the analysed species are expressed in mg of GAE or QE per mg of dry weight, as appropriate (Table 1).

The species with the greatest content of total phenols were *A. dealbata* and the two native species of the genus *Pistacea* (*P. lenticus* and *P. terebinthus*), showing double the amount of these compounds compared with *S. babylonica*, *A. negundo,* and *C. ladanifer*. Regarding flavonoids, *S. babylonica* showed the lowest amount of flavonoids, and *A. dealbata* obtained the greatest amount of these compounds. It is worth highlighting that there were significant differences in the quantified amount of flavonoids between the non-native species.

### 2.2. Bioactivity Test on Lactuca sativa

**Figure 1:**
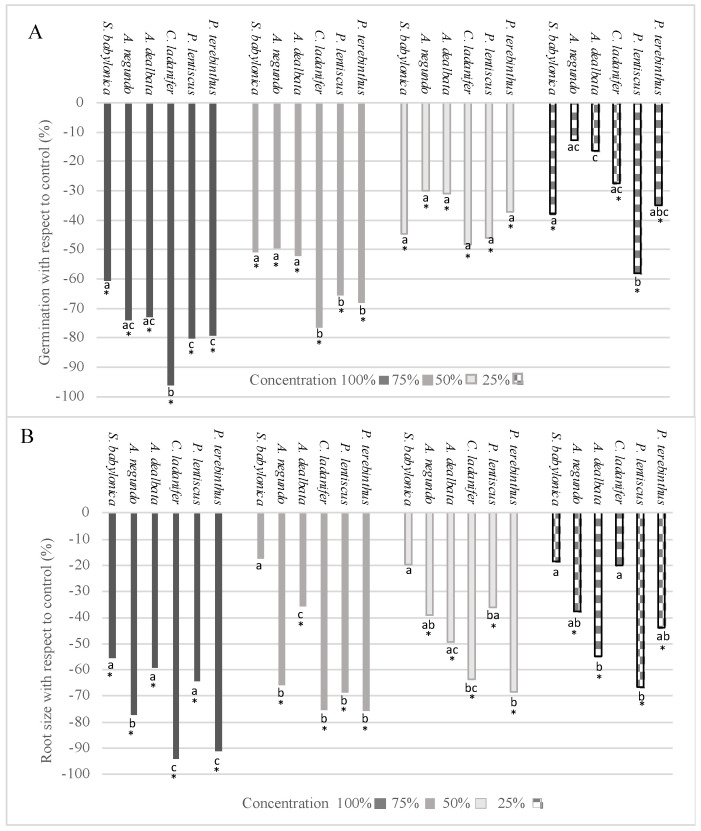
Effect of *S. babylonica, A. negundo, A. dealbata, C. ladanifer, P. lentiscus,* and *P. terebinthus* extract on germination (**A**) and root size (**B**) of *L. sativa* at 100%, 75%, 50%, and 25% concentrations; a, b, c: lowercase letters indicate significant differences between species of the same concentration (Mann-Whitney test, *p* < 0.05). *: Significant difference of control (Mann-Whitney test, *p* < 0.05).

The effect of the selected species on the percentage of germination and root size of *L. sativa* are shown in Figure 1A,B. The aqueous extracts of all 6 species significantly inhibited germination, except those from *A. negundo* and *A. dealbata* at 25%. Moreover, with the solutions at 100% and 75%, there were differences between species. At the maximum concentration, *C. ladanifer* showed the greatest negative effect, whereas at 75%, the most significant inhibition was exerted by the 3 native species.

The effect of the different extracts was more heterogeneous on the root size (Figure 1B) than on germination. With the solutions at 100% and 75%, the species that most significantly affected root size were *A. negundo*, *C. ladanifer*, and *P. terebinthus*, as well as *P. lentiscus* at 75% extract concentration. In the tests with the extracts at 50% and 25%, the inhibition exerted by each of the species was very similar, except for that of *S. babylonica*, which did not exert significant inhibition at these concentrations, as well as *C. ladanifer* at 25%. It is worth noting that the extract of *S. babylonica* only showed significant inhibition at the maximum concentration.

### 2.3. Bioactivity Test on Lavandula stoechas and Echium plantagineum

**Figure 2:**
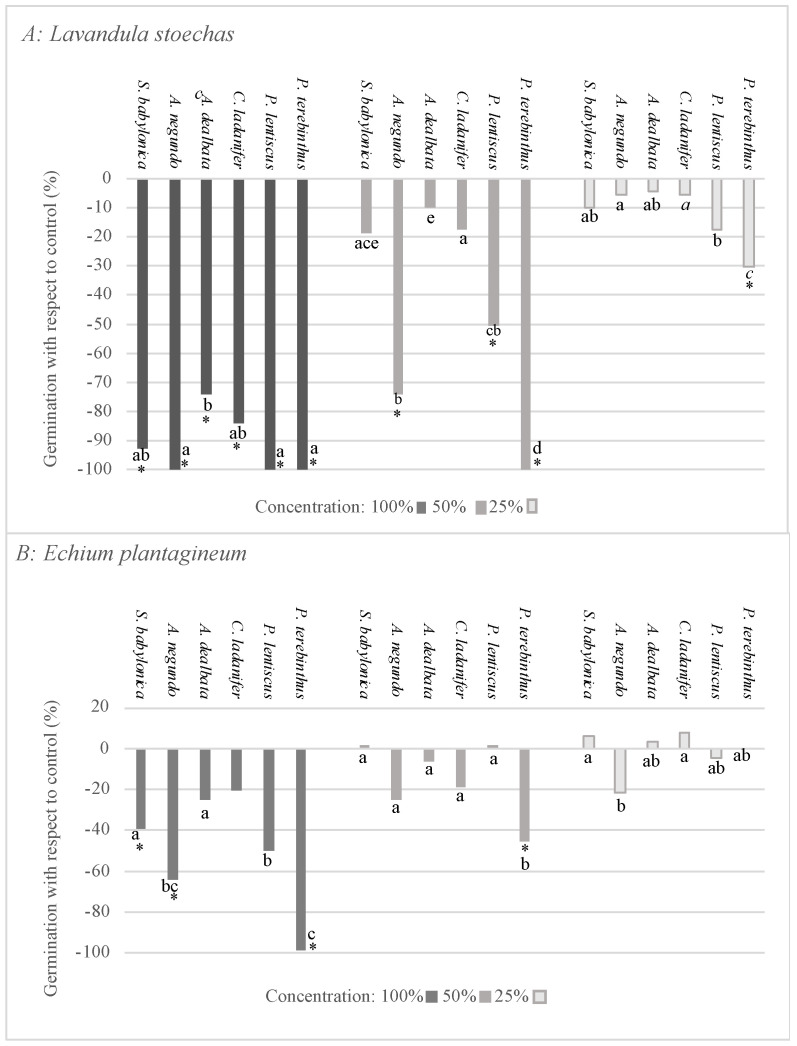
Effect of *S. babylonica*, *A. negundo*, *A. dealbata*, *C. ladanifer*, *P. lentiscus*, and *P. terebinthus* extract on germination of *L. stoechas* (**A**) and *E. plantagineum* (**B**) at 100%, 50%, and 25% concentrations; a, b, c, d, e: lowercase letters indicate significant differences between species on the same concentration (Mann-Whitney test, *p* < 0.05). *: Significant difference of control (Mann-Whitney test, *p* < 0.05).

The results derived from the germination test with *L. stoechas* and *E. plantagineum* are shown in Figure 2A,B. As can be observed, the effect on the germination of the extracts of the six analysed species differed between *L. stoechas* and *E. plantagineum*, with the seeds of target species *L. stoechas* being more sensitive to all species. The species with the greatest effect on *L. stoechas* was *P. terebinthus*, which significantly inhibited this parameter at all three concentrations tested (Figure 2A). The extracts of *A. negundo* and *P. lentiscus* exerted significant inhibition at 100% and 50%. Lastly, species *C. ladanifer* and *A. dealbata* only showed significant inhibition at the maximum concentration.

Regarding the effect on the germination of *E. plantagineum* (Figure 2B), the extracts of species *P. lentiscus*, *A. dealbata*, and *C. ladanifer* did not exert significant inhibition at any concentration; *A. negundo* and *S. babylonica* only showed significant inhibition at the maximum concentration, and *P. terebinthus* exerted significant inhibition at 100% and 50%.

**Figure 3:**
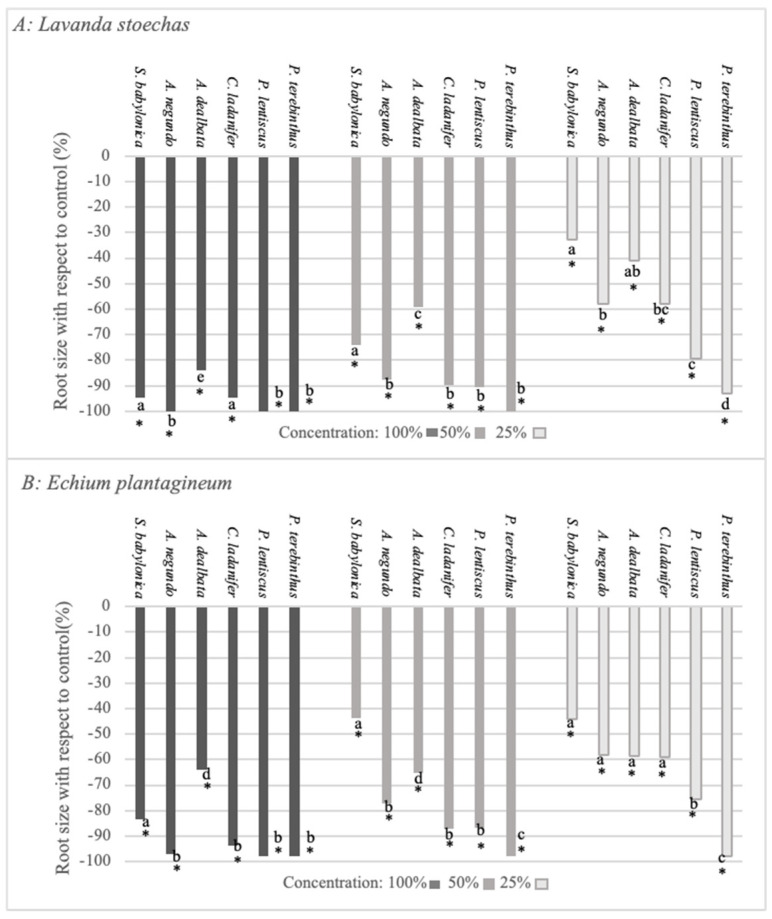
Effect of *S. babylonica*, *A. negundo*, *A. dealbata*, *C. ladanifer*, *P. lentiscus*, and *P. terebinthus* extract on root size of *L. stoechas* (**A**) and *E. plantagineum* (**B**) at 100%, 50%, and 25% concentrations; a, b, c, d, e: lowercase letters indicate significant differences between species on the same concentration (Mann-Whitney test, *p* < 0.05). *: Significant difference of control (Mann-Whitney test, *p* < 0.05).

The quantification of the effect on root size (Figure 3A,B) revealed that both *L. stoechas* and *E. plantagineum* were negatively affected by all species at all extract concentrations, although there were significant differences between species. The root size of *L. stoechas* was significantly inhibited by *A. negundo*, *P. lentiscus,* and *P. terebinthus*, and that of *E. planagineum* was significantly inhibited by the extracts of *A. negundo*, *C. ladanifer*, *P. lentiscus,* and *P. terebinthus*. It is worth highlighting that *P. terebinthus* was the species that most negatively affected both target species, whereas *S. babylonica* and *A. dealbata* showed the lowest inhibition.

**Figure 4:**
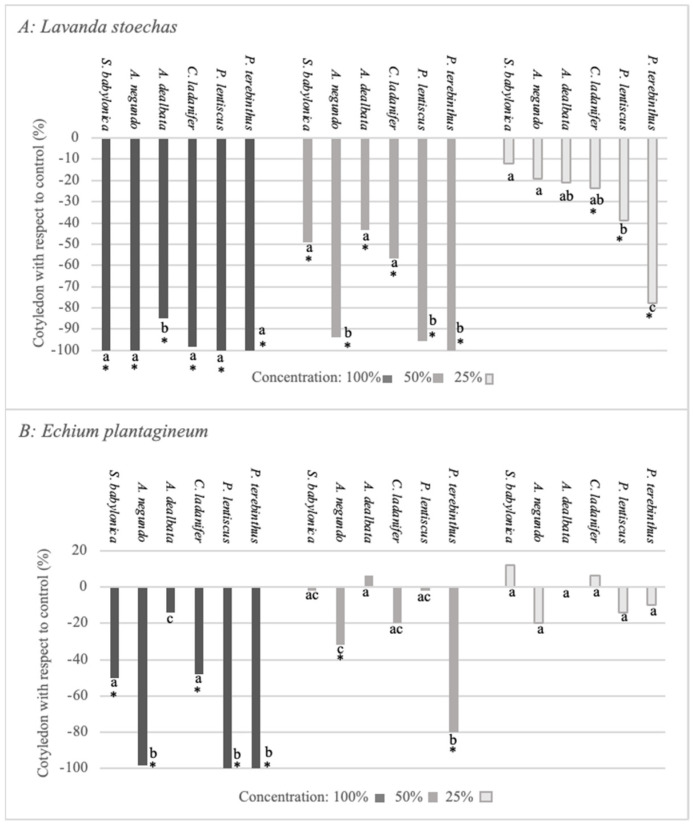
Effect of *S. babylonica, A. negundo, A. dealbata, C. ladanifer, P. lentiscus,* and *P. terebinthus* extract on cotyledon of *L. stoechas* (**A**) and *E. plantagineum* (**B**) at 100%, 50%, and 25% concentrations; a, b, c: lowercase letters indicate significant differences between species on the same concentration (Mann-Whitney test, *p* < 0.05). *: Significant difference of control (Mann-Whitney test, *p* < 0.05).

With respect to the effect caused by the extracts of these species on cotyledon emergence (Figure 4A,B), once again, *L. stoechas* was the most negatively affected, with *C. ladanifer*, *P. lentiscus,* and *P. terebinthus* maintaining a significant negative effect at the lowest concentration. On the other hand, *A. negundo* and *P. terebinthus* exerted the most negative effect on *E. plantagineum* at 100% and 50%. The lowest concentration (25%) of none of these species exerted significant inhibition on this parameter in *E. plantagineum*, and the extract of *A. dealbata* did not show significant effects at any of the analysed concentrations.

**Figure 5:**
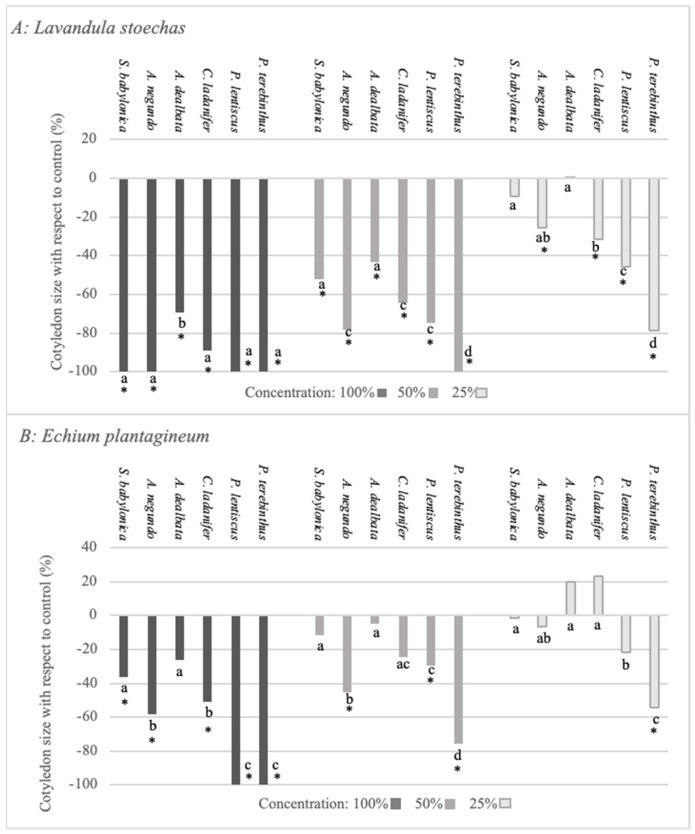
Effect of *S. babylonica, A. negundo, A. dealbata, C. ladanifer, P. lentiscus,* and *P. terebinthus* extract on cotyledon size of *L. stoechas* (**A**) and *E. plantagineum* (**B**) at 100%, 50%, and 25% concentrations; a, b, c, d: lowercase letters indicate significant differences between species on the same concentration (Mann-Whitney test, *p* < 0.05). *: Significant difference of control (Mann-Whitney test, *p* < 0.05).

Another morphological parameter quantified was cotyledon size (Figure 5A,B). As can be observed, these species exerted a negative effect on the growth of *L. stoechas* and *E. plantagineum*. In *L. stoechas*, the significant negative effect was maintained for *A. negundo, C. ladanifer, P. lentiscus*, and *P. terebinthus* at the lowest concentration, with the extracts of *S. babylonica* and *A. dealbata* showing no activity at this concentration. It is important to mention that *P. terebinthus* had the greatest negative effect, and *S. babylonica* and *A. dealbata* were the species with the lowest negative effect. The negative effect observed on cotyledon size in *E. plantaginum*, as well as for the rest of the parameters, was lower than that produced in *L. stoechas*. At the lowest extract concentration, only *P. terebinthus* maintained the negative effect. It is worth pointing out that *A. dealbata* did not have a negative effect at any of the analysed concentrations. 

Comparing the effect of the native species, as a group, with that of the non-native species (Table 2), the group of native species produced the greatest percentages of inhibition in the four parameters analysed in both target species. However, no significant differences were observed in germination, but differences were observed in root size, cotyledon emergence, and cotyledon size. For *L. stoechas*, there were significant differences between these 2 groups of species in root size and cotyledon emergence with extracts at 50% and 25%, and with 25% in cotyledon size. In *E. plantagineum*, the native species exerted a more significant inhibition on root size at all 3 concentrations, on cotyledon emergence at maximum concentration, and on cotyledon size at 100% and 25%.

## 3. Discussion

To better understand the process of the invasive species, it is fundamental to analyse their colonisation strategies, especially their allelopathic potential [26]. Allelopathy is an interaction that involves secondary metabolites, mostly phenols, thus knowing the amount of total phenols and flavonoids present in these species is an initial and necessary process [37].

Great variability was observed in the amount of total phenols and flavonoids among the selected species in the present study, ranging from 0.026 to 0.010 mg QE/mg of dry weight for phenols, and from 0.016 to 0.004 mg QE/mg of dry weight for flavonoids. The species with the greatest amounts of total phenols and flavonoids were *P. terebinthus*, *A. dealbata*, and *P. lentiscus*, which have been classified as phytotoxic by other authors [48,49,50,51,58,67].

The bioassays with *L. sativa* showed that both the non-native and native species exerted allelopathic activity, although the effect on germination and root development was different between them. Among the native species, the greatest negative effect, for both germination and root size, was shown by *P. lentiscus* and *P. terebinthus*, and among the non-native species, *Salix babylonica* exhibited the greatest negative effect on germination; on the other hand, root size was most significantly affected by *A. negundo* and *A. dealbata*. It was observed that the native species with the greatest phenol content were the ones with the greatest allelopathic potential; however, in the non-native species, this only applied to the effect of *A. dealbata* on root size. It is important to mention that the phytotoxicity of *A. negundo* quantified in previous studies [61] depends on the extract concentration, with the negative effect on germination and root size disappearing at lower concentrations. In our study, this behaviour was observed on germination, but not on root size.

Therefore, both the non-native species and the native species show allelopathic potential, although their effects may differ when acting on native species. In the bioassays conducted with *E. plantagineum* and *L. stoechas*, it was revealed that, firstly, the sensitivity to the extracts of different species is different in the two target species, with a more significant negative effect on the germination, cotyledon emergence, and cotyledon size of *L. stoechas* than on those of *E. plantagineum*. The different behaviour of the extracts based on the target species has been reported in previous studies [68,69,70], with our results confirming the need to use different target species to study the allelopathic behaviour of a specific species. Secondly, our study also corroborates that root size is the most sensitive morphological parameter in the evaluation of the allelopathic activity of a species [71]. As can be observed, root size was negatively affected in *L. stoechas* and *E. plantagineum* by the native species, and not the non-native species, at all three concentrations.

The results also show that *P. terebinthus* exerted the greatest negative effect. This was the only species that maintained a significant effect at 50% and 25% in all the quantified parameters, except on germination and cotyledon emergence in *E. plantagineum* at the lowest concentration. Moreover, the comparison between the group of native species and the group of non-native species (Table 2) revealed that there were no significant differences in the effect on germination, although there were significant differences in the effect on root size at all three concentrations, with the native species exerting the greatest inhibition. It is worth highlighting that, although all species significantly inhibited root size, *P. lentiscus* and *P. terebinthus* produced the greatest negative effect on both target species. These results show that the inhibitory effect of the non-native species was significant but native species had a greater effect. This indicates that the non-native species do not have allelopathic compounds that are more active on the native species than the autochthonous species of this community, as opposed to the NWH [32]. Similar results were found in other studies such as those conducted by Corina Del Fabbro [72], who analysed and compared the allelopathic activity of invasive species of Central Europe with that of native species of these communities, concluding that the effect on the target species selected was similar in both groups. Similarly, De las Heras et al. [73] studied the phytotoxicity of invasive species *Ailanthus altissima*, *Robinia pseudoacacia*, and *Ulmus pumila*, and native species *Populus alba*, *Populus nigra*, and *Ulmus minor*, finding that the negative effect on the three target species selected was similar.

On the other hand, there are studies that report that the effect produced by invasive species is greater than that of native species [35,36,37,38,74]. The study conducted by Kim and Lee [37] with invasive plants from Eastern Asia shows that the invasive species produced more phenols and were more allelopathic than the native species of Korea.

These controversial and apparently contradicting results may be dependent on habitat and characteristics of the ecosystems. For example, the arid and semiarid climatic conditions of Mediterranean ecosystems subject native species to great abiotic stress [75], leading these species to exhibit a great diversity of secondary metabolites that are potentially allelopathic [76]. In these ecosystems, allelopathy is a frequent interaction between species [22,75,76,77], which would explain the absence of differences between invasive and non-invasive species, as well as the greater effect shown by the native species in our study. On the contrary, in other ecosystems in which the environmental conditions are less stressful, native species have a smaller amount of allelopathic compounds, as in the study conducted by Kim and Lee [37] in Korea. It would be interesting to carry out further studies to compare the allelopathic activity of native and non-native species from different types of ecosystems, in order to determine whether the characteristics of the ecosystem influence the greater or lesser activity that non-native species may show compared with native species. 

It should be noted that the results of this work are derived from bioassays and that a better understanding of the allelopathic effect of natives and non-natives species will require further studies in the field. In addition, it would be interesting to study whether non-native species are sensitive to native allelopathic species, which could contribute to a natural method of invasive species control [78,79]. These studies can help to understand the integrative role of allelopathy in plant invasion. 

## 4. Materials and Methods

### 4.1. Gathering of Materials and Sample Treatment

The samples were gathered in the province of Badajoz (Spain) (38°52′43” N, 6°58′13” W), except for *P. terebinthus*, which was obtained in the mountain range of Montánchez, in the province of Cáceres (Spain) (39°15′ N, 5°58′ O). In September 2021, approximately 500 g of leaves of each of the species were gathered. Samples were collected from different individuals, which were randomly selected. 

The samples were taken to the laboratory on the same day as the sampling was conducted, and they were left to dry at room temperature. 

When the leaves were dry enough, they were ground by hand until pieces of 1–2 cm^2^ were obtained and were then kept in the dark at room temperature. 

### 4.2. Preparation of the Aqueous Extracts 

Aqueous solutions were prepared to carry out the allelopathic analyses. Dry leaves were mixed with distilled water (1:10 *w/v*) and were left stirring at room temperature for 24 h. Then, the samples were filtered with filter paper and 4 concentrations of the aqueous extracts of each species were prepared. To this end, the original solution (100%) was diluted with distilled water to obtain concentrations of 75%, 50%, and 25%.

### 4.3. Extraction and Quantification of Total Phenols 

For the extraction of total phenols, the leaves were ground in a mechanic grinder until a fine power was obtained, and then methanol was added (1:10 *w/v*). It was left stirring for 24 h at room temperature. After that, they were centrifuged at 4500 rpm for 15 min and, once the supernatant was evaporated, it was re-suspended in methanol at a concentration of 1 mg/mL [80].

The total phenolic content was calculated through the Folin–Ciocalteu reagent test, following the method of Singleton and Rossi [81]. A 1 mL aliquot of the extract diluted in methanol (1 mg/mL, 3 replicates per sample) was taken, to which 500 μL of Folin–Ciocalteu reagent and 6 mL of distilled water were added. The mix was left stirring for 5 min and, after that time, 1.5 mL of Na_2_CO_3_ (20%) and 1.9 mL of distilled water were added while stirring to homogenise the dilution. After incubation in the dark for 2 h, absorbance was measured at 760 nm in a UV-30 spectrophotometer. The blank was obtained by replacing the amount of diluted extract with methanol. 

The results are expressed in mg of gallic acid equivalents (GAE) per mg of dry weight and mg of quercetin equivalents (QE) per mg of dry weight. 

### 4.4. Extraction and Quantification of Total Flavonoids 

The extraction of total flavonoids was carried out following the same procedure as for the extraction of phenols, although the sample was re-suspended in ethanol at 80% at a concentration of 1 mg/mL. 

The total flavonoids were quantified following the method proposed by Chang et al. [82]. Firstly, 2 mL of ethanol were taken, to which we added 0.5 mL of extract (1 mg/mL re-suspended in 80% ethanol, 3 replicates) and 0.15 mL of NaNO_2_ (1 M in ethanol). The mixture was stirred and left to settle for 3 min. Then, 0.15 mL of AlCl_3_ (10%, in ethanol) was added, and the sample was stirred again for 3 min. Subsequently, we added 1 mL of NaOH (1 M in distilled water) and ethanol to 5 mL of total volume. 

The solutions were mixed again after the last steps and kept in the dark for 40 min. Then, absorbance was measured at 415 nm using a UV-30 spectrophotometer. The blank was obtained by replacing the amount of diluted extract with ethanol. 

The results are expressed in mg of quercetin equivalents (QE) per mg of dry weight.

### 4.5. Bioassays 

#### 4.5.1. Bioassays on *Lactuca sativa*

The allelopathic potential of the 6 species selected was quantified using *L. sativa* as the target species. This species is considered a comparable indicator of allelopathy among species due to its rapid germination and for being commonly used in phytotoxic studies [83,84]. 

A total of 25 seeds were placed in Petri dishes with filter paper (4 replicates for each concentration). Then, to each dish 5 mL of the dilution was added, and the dishes were sealed with Parafilm. A total of 10 dishes were prepared for the control, following the same procedure, although adding distilled water instead of the dilution. 

After preparation, the dishes were randomly placed in the culture chamber. They were kept at 20 °C with a photoperiod of 16 h of light and 8 h of darkness for 10 days. After this time, the amount of germinated seeds and seedlings with cotyledons was quantified in each dish. Similarly, 10 seedlings per treatment were randomly selected, and their radicle and cotyledon length was measured. 

The results are expressed in percentage with respect to the control. 

#### 4.5.2. Bioassays on *Echium plantagineum* and *Lavandula stoechas*

Bioassays were conducted on the native species *E. plantagineum* and *L. stoechas* with the extracts of the 6 species selected. *E. plantagineum* and *L. stoechas* were selected for being common species in the Iberian Peninsula and for sharing the habitat with native species [65,66]. The seeds were purchased from Semillas Silvestres S.L. (http://www.semillassilvestres.com, accessed on 13 February 2023).

For the germination and seedling growth bioassays, the same procedure used with *L. sativa* was followed, although only 3 concentrations of aqueous extract were used: 100%, 50%, and 25%. 

The results of the variables germination, root size, and cotyledon size are expressed in percentage with respect to the control. 

### 4.6. Statistical Analysis 

For the data treatment, we used the mean values of the different measured variables. For the data analysis, we used the R programme (with the R Commander package). Firstly, we analysed whether all the measured variables followed a normal distribution, using the Shapiro–Wilk test. The variables flavonoids (*p*-value = 0.4017), germination in *L. sativa* (*p*-value = 0.44), root size in *L. sativa* (*p*-value = 0.15), cotyledons in *E. plantagineum* (*p*-value = 0.15), germination in *E. plantagineum* (*p*-value = 0.925), and cotyledon size in *E. plantagineum* (*p*-value = 0.572) showed normal distribution, and their homoscedasticity was determined using Bartlett’s test for homogeneity of variances. The variable flavonoids (*p*-value = 0.2878) showed homoscedasticity. Since the variable flavonoids followed a normal distribution and its variance was constant, an ANOVA test was used to compare the means of the species, and Tukey’s test was used for the pairwise comparison of means. For the rest of the variables, the Kruskal–Wallis non-parametric test was used to compare the means, and the Mann–Whitney test was used for the pairwise comparison of means. Statistical significance, in all cases, was established at *p* < 0.05. 

## 5. Conclusions

In conclusion, our results corroborate that both the non-native species and the native species studied here are allelopathic. These species exert a negative effect on the selected native target species, but on some of the measured morphological parameters, the negative effect of the native species is greater than that of the non-native species, in contradiction to the predictions of the novel weapons hypothesis. It highlights the importance of comparing allelopathic effects of invasive and native species to correctly estimate the phytotoxic effect of non-native species on their invasiveness. 

## Figures and Tables

**Table 1:** Total phenols and flavonoids in the extracts of selected species. *n* = 3.

	Phenol Total		Flavonoids
	GAE (mg/mg dw)	EQ (mg/mg dw)	EQ (mg/mg dw)
*S. babylonica*	0.010 ^a^	0.008 ^a^	0.004 ^a^
*A. negundo*	0.012 ^a^	0.010 ^a^	0.008 ^b^
*A. dealbata*	0.025 ^b^	0.021 ^b^	0.016 ^c^
*C. ladanifer*	0.013 ^a^	0.012 ^a^	0.010 ^b^
*P. lentiscus*	0.024 ^b^	0.020 ^b^	0.012 ^c,b^
*P. terebinthus*	0.026 ^b^	0.022 ^b^	0.012 ^c,b^

GAE: gallic acid equivalents; QE: quercetin equivalents; dw: dry weight; ^a, b, c^: lowercase letters indicate significant differences between species (Mann-Whitney test to phenols total, *p* < 0.05; Tukey test to flavonoids, *p* < 0.05).

**Table 2:** Percentage on germination, root size, cotyledon emergence, and cotyledon size according to the group of species and extract concentration in *E. plantagineum* and *L. stoechas*. The values are the mean of the species that constituted the native group and the non-native group as an activity function with respect to the control (negative values mean inhibition; positive values mean stimulation).

		*Lavandula stoechas*			
Extract	Species	Germination	Root Size	Cotyledon	Cotyledon Size
**100%**	Native	−94.68 ^a^	−98.20 ^a^	−99.50 ^a^	−96.36 ^a^
	Non native	−88.88 ^a^	−92.83 ^a^	−95.02 ^a^	−89.82 ^a^
**50%**	Native	−56.03 ^a^	−93.40 ^a^	−84.07 ^a^	−79.68 ^a^
	Non native	−34.29 ^a^	−73.69 ^b^	−62.18 ^b^	−57.93 ^a^
**25%**	Native	−17.87 ^a^	−76.74 ^a^	−46.76 ^a^	−51.93 ^a^
	Non native	−6.76 ^a^	−43.86 ^b^	−17.41 ^b^	−11.44 ^b^
		***Echium plantagineum***			
		**Germination**	**Root size**	**Cotyledon**	**Cotyledon size**
**100%**	Native	−56.25 ^a^	−96.54 ^a^	−82.66 ^a^	−83.57 ^a^
	Non native	−42.70 ^a^	−81.47 ^b^	−54.00 ^b^	−40.14 ^b^
**50%**	Native	−20.83 ^a^	−90.51 ^a^	−34 ^a^	−43.42 ^a^
	Non native	−9.89 ^a^	−62.04 ^b^	−9.33 ^a^	−20.69 ^a^
**25%**	Native	1.04 ^a^	−77.47 ^a^	−6.00 ^a^	−17.69 ^a^
	Non native	−4.16 ^a^	−53.9 ^b^	−2.66 ^a^	3.91 ^b^

^a, b^: Lowercase letters indicate significant differences between native and non-native species (Mann-Whitney test, *p* > 0.05).

## Data Availability

Not applicable.
